# Potential Mechanisms of Quercetin Influence the ClfB Protein During Biofilm Formation of *Staphylococcus aureus*

**DOI:** 10.3389/fphar.2022.825489

**Published:** 2022-01-28

**Authors:** Xinyun Kang, Qiang Ma, Guilai Wang, Na Li, Yanni Mao, Xin Wang, Yuxia Wang, Guiqin Wang

**Affiliations:** ^1^ Veterinary Pharmacology Lab, School of Agriculture, Ningxia University, Yinchuan, China; ^2^ Yinchuan Hospital of Traditional Chinese Medicine, Yinchuan, China

**Keywords:** *Staphylococcus aureus*, biofilm, clfB, quercetin, inhibition

## Abstract

This study aimed to establish the mode of binding between Quercetin (QEN) and an essential protein called ClfB in forming biofilm in *Staphylococcus aureus* (*S. aureus*). In this study, the raw data of GSE163153 were analyzed for quality control, alignment, and gene counts, and the differential analysis detected the key differentially expressed genes (DEGs) assisting in the formation of the *S. aureus* biofilm. Then, the protein-protein interaction (PPI) and gene function enrichment analyses of the target genes, identified a gene called *clfB* to be closely related to biofilm formation. ClfB was structurally characterized, molecularly docked, and kinetically simulated to unravel the mode of binding of QEN to ClfB. Meanwhile, the growth curve and transmission electron microscopy methods examined the effect of QEN on the *S. aureus* growth. Results indicated that the *clfB* gene was increasingly expressed during biofilm formation and was involved in cell adhesion, pathogenicity, and infection. We identified 5 amino acid sites of ClfB (D272, R331, I379, K391, E490) as potential sites for binding QEN, which would indirectly influence the changes in the functional sites N234, D270, Y273, F328, inhibiting the formation of biofilm. Meanwhile, 128 μg/ml of QEN could significantly inhibit the *S. aureus* biofilm formation. This manuscript serves as a molecular foundation for QEN as an antibacterial drug providing a new perspective for developing antibacterial drugs.

## Introduction

*Staphylococcus aureus* (*S. aureus*) is a Gram-positive bacterium infecting humans and animals, existing either as unicellular or sessile aggregates (known as biofilms). Compared to the planktonic cells, the biofilm bacteria cells are more resistant to most antibiotics and host defenses (Nostro et al., 2017) and more adaptable to diverse conditions (Stewart et al., 2001; Gil, 2014). Due to this characteristic, antibiotics fail to diffuse effectively into the peritrichous matrix and cannot easily change the cellular growth state of peritrichous bacteria, producing peritrichous populations that resist high levels of antibiotic tolerance with slow growth. Almost 60% of *S. aureus* infections are triggered due to biofilm formation (Costerton, 1999). The establishment of *S. aureus* biofilm leads to a resistance to antimicrobial therapy and host immune response. As a result, it imparts the pathogen with the ability to trigger recurrent infections, leading to the ineffectiveness of a single antimicrobial drugs administration (Costerton, 1999). The *β*-lactams, fluoroquinolones, and aminoglycosides are conventionally used for treating biofilm infections (Smith et al., 2009; Zimmerli, 2014). In addition to the pharmacology, the treatment strategies focus on two aspects: The antimicrobial coatings, anti-adhesive surfaces, and vaccines preventing the biofilm formation before the growth of the bacteria (Fu et al., 2005). Upon biofilms infecting the organisms, the methods such as matrix-degrading enzymes, dispersion triggers, small molecule inhibitors, and network modulation are employed for targeting the infection (Park et al., 2007; Chung and Toh, 2014; Chung et al., 2018). In fact, the widespread use of antibiotic therapy renders the therapeutic efficiency of antibiotics incapable of keeping up with the rate of the strain mutation (Parsek and Singh, 2003; Mah, 2012; Holder et al., 2013). Once the biofilm is established, the bacteria within the biofilm require more antibiotics than those required by the planktonic bacteria (Wu et al., 2015; Memariani et al., 2019). As a result, it leaves humans at risk from an ever-increasing number of antibiotics.

Quercetin (QEN) is a plant metabolite, a polyphenolic flavonoid found widely in fruits, vegetables, nuts, seeds, bark, flowers, and leaves (Williamson and Manach, 2005; Wiczkowski et al., 2008). The available data indicate QEN significantly inhibited the *S. aureus* biofilm formation (Lee et al., 2013), but studies obtained various MICs (Cho et al., 2015; Júnior et al., 2018). Compared to the other flavonoids, QEN with five phenolic hydroxyl groups has the most substantial inhibitory effect (Cho et al., 2015). It is capable of inhibiting the expression of biofilm-related genes such as adhesion-related genes *icaA* and *icaD* (O'Gara, 2007), the quorum-sensing-related gene *agrA* (Markowska et al., 2013), and virulence-regulated genes *sigB* and *sarA* (Lee et al., 2013). This paper intends to discover the inhibition mechanism of QEN on the biofilm formation of *S. aureus* based on the limitations of the mentioned research advances and the diversity and widespread features of QEN, using bioinformatics, molecular dynamics, and experimental validation to find new targets and to provide novel perspectives for treating *S. aureus* infection.

## Materials and Methods

### Bacterial Strain

This study used standard bacterial strains *S. aureus* like the *ATCC33591*, *ATCC29213*, and *WLD10*, *XF6*, *LN25*, and *JY45*. All these bacterial strains were maintained in our laboratory.

### Culture Medium and Reagents

The MH broth, MH agar, and TSB broth were purchased from Beijing Luqiao Technology Co., Ltd. The Crystal violet was acquired from Sigma-Aldrich, United States, while the absolute ethanol and acetone were bought from Sinopharm Chemical Reagent Co., Ltd.

### Equipment

The equipment like UV spectrophotometer (Beckman, United States), ultra-clean bench (Shanghai Boxun Industrial Co., Ltd. Medical Equipment Factory), transmission electron microscope (Hitachi/HITACHI, Japan), diamond slicer (Daitome/Ultra45°, United States), a gel imaging system (Bio-Rad, United States) and the Ultra-thin slicer (Leica/LeicaUC7, United States) were used.

### Data Preparation

Integrating public data resources is invaluable to reusing and uncovering data’s potential value from distinct standpoints. The GSE163153 dataset (Tomlinson et al., 2021) (https://www.ncbi.nlm.nih.gov/geo/query/acc.cgi?acc=GSE163153) is used here was downloaded from the GEO database (https://www.ncbi.nlm.nih.gov/geo/). Briefly, this dataset contains the transcriptome datasets of five bacterial strains *S. aureus* (*N315*, *MRSA252*, *LAC*, *MW2*, and *NRS385*) at 5, 10, and 24 h biofilm, with 4, 4, and 8 samples of the five bacterial strains at 5, 10, and 24 h, respectively. The *S. aureus* genome and annotation files were downloaded from the Ensembl Bacteria (http://bacteria.ensembl.org/), and the genome index was constructed using the Hisat2 software (version 2.2.1, http://daehwankimlab.github.io/hisat2/). After downloading the raw data, the global data quality was evaluated using the FastQC (Kassambara, 2017) (version 0.11.7, https://www.bioinformatics.babraham.ac.uk/projects/fastqc/) software with a quality score threshold of Q20 (false discovery rate, FDR < 0.01). The data were quality-controlled using the Trim-galore (version 0.6.6, https://www.bioinformatics.babraham.ac.uk/projects/trim_galore/) software to remove the adapters, low quality, and Poly-N sequences to ensure that the quality score of all the samples was greater than Q20. The clean reads were aligned to the *S. aureus* genome using the HISAT2 software (version 2.2.1, http://daehwankimlab.github.io/hisat2/) to obtain the *sam* files containing the alignment information, and the sam files were subsequently converted to the bam files using the Samtools (Li et al., 2013) (version 1.9, https://sourceforge.net/projects/samtools/files/samtools/1.9/) software. The data were processed using the FeatureCounts (version 2.0.1, http://subread.sourceforge.net/) software in the subread package (Kim et al., 2016) to count the *bam* files at the gene level and obtain the expression matrix for each sample. TPM (Transcripts Per Kilobase of exon model per Million mapped reads) is an excellent method for quantitating RNA abundance and is proportional to the average relative RNA molar concentration. Many computational algorithms use TPM for transcript quantification (e.g., RSEM(Dewey and Li, 2011) and Salmon (Roparo et al., 2017) methods), thereby we use TPM for gene quantification. The formula for TPM is 106× [(reads mapped to transcript÷transcript length) ÷ Sum (reads mapped to transcript÷transcript length)].

### Differential Expression Analysis

The above steps yielded a matrix of counts that satisfied the conditions for investigating the genes associated with biofilm formation at different times. The five bacterial strains *S. aureus* (*N315*, *MRSA252*, *LAC*, *MW2*, and *NRS385*) were combined into one treatment at the same period for differential analysis at 5 h (n = 20) vs. 10 h (*n* = 20) and 10 h (*n* = 20) vs. 24 h (*n* = 40) after obtaining the count matrix. The genes expressed at the same period were also differentially expressed for both the planktonic and biofilm bacteria. The differentially expressed genes (DEGs) were screened using the R package DESeq2 (version 1.24.0) with a threshold of fold change (FC) ≥ 2 and false discovery rate (FDR) < 0.05 (Love et al., 2014).

### Functional Annotation of the Key Genes and Analysis of the Protein Interaction Networks

Gene function is primarily dependent on the function of its coded protein, and genes with similar structures are functionally similar. The protein interaction network of the differential expressed genes were obtained using the STRING database (https://string-db.org/, version 11.0) and subsequently visualized using the Cytoscape software (https://cytoscape.org/, version 3.6.1). The topology calculations were performed using the Network Analyzer tool of the Cytoscape software, and the top 10 genes were selected as the key genes in terms of the degree values. These ten genes were annotated functionally and per the pathway on the DAVID website (https://david.ncifcrf.gov/). For functional annotation (Gene Ontology), the cellular component (CC), molecular function (MF), and biological process (BP) were considered, followed by the pathway annotation (Kyoto encyclopedia of genes and genomes, KEGG).

### Structural Characterization of the ClfB Protein

It is essential to estimate the structure of a protein to understand its function. The ClfB protein loci were analyzed using the online servers, ESPript3.x (https://espript.ibcp.fr/ESPript/ESPript/) and New ENDscript 2 Server (http://endscript.ibcp.fr). The conservation of amino acid residues of ClfB was analyzed using the server Consurf Web Server (https://consurf.tau.ac.il/), and the PyMOL software (https://pymol.org/2/, version 2.5) was used to correlate it with the structure and function of ClfB protein by mapping it to the tertiary structure of the ClfB protein.

### Molecular Docking

The PDB file for ClfB (PDB ID: 4F1Z) was downloaded from the RCSB database (https://www.pdbus.org/) as the receptor protein; the QEB molecular structure was downloaded from the PubChem database (https://pubchem.ncbi.nlm.nih.gov/) for energy minimization [based on the MMFF94 forcefield (Halgren, 1996)] to generate a structure file that could be adapted for molecular docking. The receptor protein ClfB was followed by hydrogenation using AutoDockTools 1.5.6 software; the number of rotatable bonds and the Grid Box of the ligand molecule were set. Semi-flexible molecular docking of the receptor proteins was performed using the AutoDock Vina and Smina programs (Trott et al., 2009) (the results obtained were confirmed by the consistency of the two programs mentioned above). After these steps, the appropriate scores and conformation results were selected and visualized using PyMOL (https://pymol.org/2/) and Discovery Studio 2020.

### Molecular Dynamics Simulation

Using the classical molecular dynamics simulation software GROMACS 2019.06 (Mja et al., 2015), all-atom molecular dynamics simulations were performed using the docked ClfB-QEN complex as the initial conformation to analyze the mechanism of interaction and to validate the reliability of the binding pattern. Both the receptor protein and the ligand molecule were used with Amber 99SB-ILDN forcefield parameters, and the ligand topology file was generated by the Antechamber and ACPYPE programs. A dodecahedral solvation box was selected to set the nearest distance between the system boundary and the complex to 1.0 nm. The TIP3P water model was selected, and Na or Cl was then randomly added to the compound system according to the VERLET cut-off method to counterbalance the protein charge. The system energy minimization, NVT temperature control, and NPT pressure control were applied to keep the system temperature at 300 K and the pressure constant at 101.325 kPa, and a 100 ns free kinetic simulation was executed on the above equilibrium system (Ebadi and Razzaghi-Asl, 2013). The Root Mean Square Deviation (RMSD) was used to represent the stability of the protein structure and the complex, while the level of structural variability as indicated by the Root Mean Square Fluctuation (RMSF) and the Radius of gyration (Rg). The number of hydrogen bonds formed between the receptor protein and the ligand molecule was analyzed concerning the simulation time.

### Growth Curve Determination of the Bacterial Strains *Staphylococcus aureus* by Quercetin

Prior to determining whether QEN affected *S. aureus* growth, available literature revealed that the MIC of QEN on *S. aureus* growth was greater than 594 μg/ml (Kuo, 2013; Perumal et al., 2017). Therefore, we established 0 μg/ml, 64 μg/ml, 128 μg/ml, 256 μg/ml, 512 μg/ml concentrations gradient to examine their MIC value. The single colonies were picked in 2 ml MHB medium and incubated at 37°C, 220 r/min overnight. The subsequent experiments were performed when the OD_600_ = 0.1 (1 × 10^8^ CFU/ml). The appropriate amount of culture medium and 100 uL of the bacterium solutionwas added to each group. Then, 50 μL of the bacterial solution was diluted to 10^3^ CFU/ml at an interval of 2 hours and dispersed on an MHA agar plate medium and incubated at 37°C for 16 h. The counting of the growing colonies followed them.

### Transmission Electron Microscopy Experiments

The *S. aureus* stock was inoculated into the MHB medium at 220 r/min and incubated at 37°C until the logarithmic growth phase, and then centrifugation at 12,500 r for 2 min. The supernatant was discarded and eluted with 1 ml PBS to prepare the transmission electron microscopy samples, observing and capturing the photographs under transmission electron microscopy.

### The Effect of Quercetin on Biofilm Formation in *Staphylococcus aureus*

Prior to determining whether QEN affected *S. aureus* biofilm formation, available literature revealed that the MBIC of QEN on the biofilm ranged from 9 μg/ml to 60 μg/ml (Amin et al., 2015; Júnior et al., 2018; Mohamed et al., 2020). Therefore, we established 0 μg/ml, 8 μg/ml, 16 μg/ml, 32 μg/ml, 64 μg/ml, 128 μg/ml concentrations gradient to examine their MBIC value *. S. aureus* was inoculated into the MHB medium and incubated at 220 r/min and 37°C until the logarithmic growth phase was reached. It was then centrifuged at 12,500 r for 5 min, and 3 ml of TSB-g medium was added after discarding the supernatant. A 24-well plate was used to prepare biofilm, and 400 μL of the bacterial solution was added to each well, followed by 1,600 μL of fresh TSB-g medium containing the drug added to make the final concentrations of QEN to 8 μg/ml, 16 μg/ml, 32 μg/ml, 64 μg/ml, and 128 μg/ml, respectively. Meanwhile, a TSB-g medium without QEN was used as a blank control and incubated at 37°C for 40 h. After discarding the floating bacteria, the crystal violet staining was performed, and the photographs were captured using a gel imaging system (Bio-Rad, United States), and absorbance at 570 nm was measured using a microplate reader.

### Statistical Analysis

All the statistical analyses were carried out using R language programs, and a paired sample *t*-test (*t*-test function) was used for statistical analysis; the significance levels were set as *p* < 0.05.

## Results

### Data Quality Control and Alignment

High-quality clean reads are essential for the accuracy of the results. The results showed that the quality scores of all the positions of clean reads for all the samples were greater than 30, i.e., FDR < 0.001, which met the subsequent analysis. By comparing the clean reads to that of the *S. aureus* genome, the percentages of 5, 10, and 24 h aligned with the genome as 90.30, 93.93, and 92.24%, respectively. The percentages with unique matching positions were 87.59, 90.59, and 89.59%, while the percentages with multiple alignment positions were 1.50, 3.17, and 2.93%, respectively (Table 1). Therefore, the alignment results indicated that the clean reads obtained from sequencing had a high probability of being derived from *S. aureus*, and the method used was reasonable.

**TABLE 1:** Alignment statistics of the clean reads.

Time (h)	Aligned (%)	Mapped uniquely (%)	Multi-mapped (%)	Neither mate aligned (%)
5	90.30	87.59	1.50	10.89
10	93.93	90.59	3.17	6.24
24	92.24	89.11	2.93	7.99

### Analysis of Differentially Expressed Genes

**FIGURE 1:**
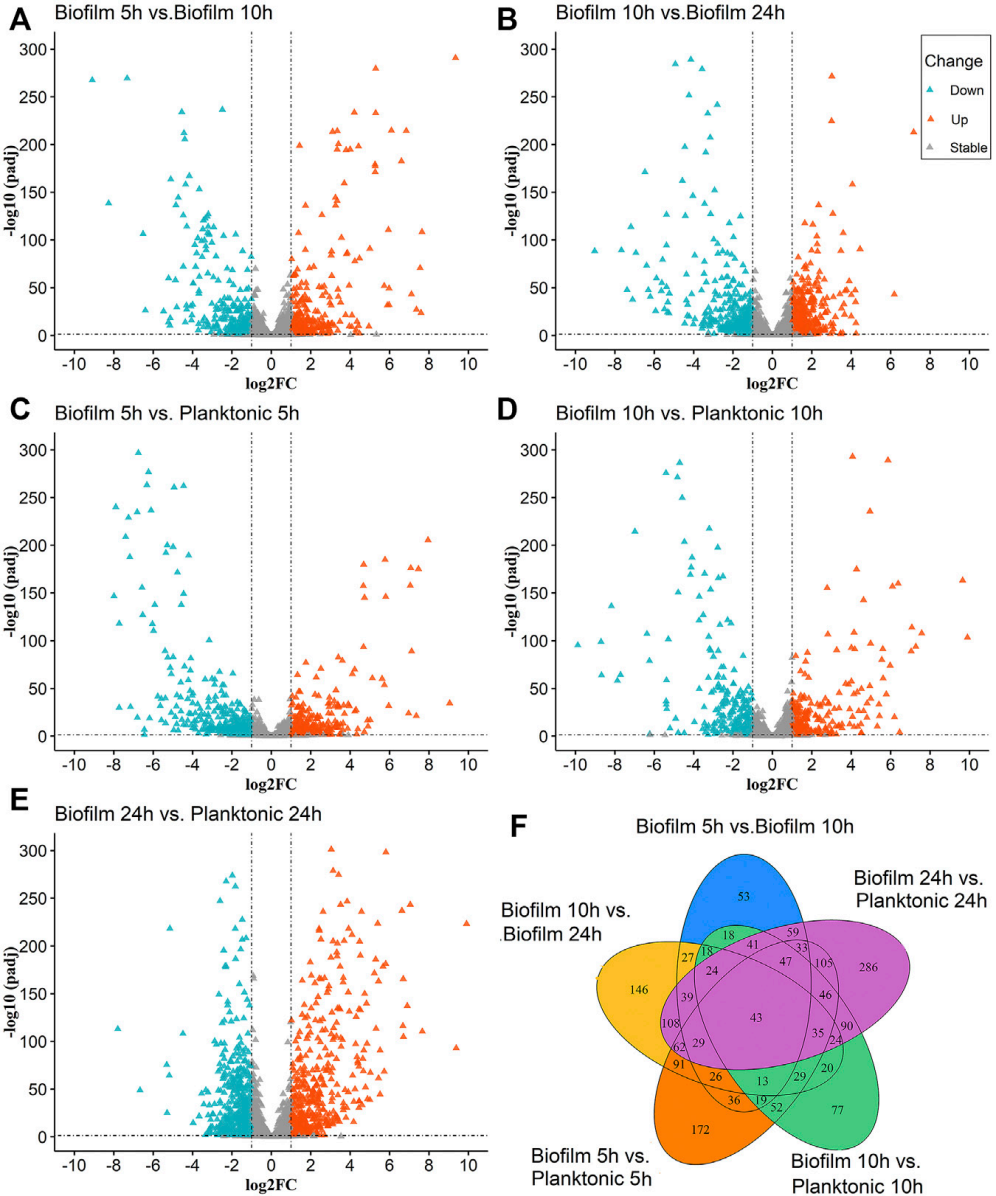
The Volcano and Venn diagrams for the differential analysis of the biofilm and planktonic bacteria; **(A–E)** indicated the differential analysis for five groups (biofilm 5 h vs. biofilm 10 h, biofilm 10 h vs. biofilm 24 h, biofilm 5 h vs. planktonic 5 h, biofilm 10 h vs. planktonic 10 h and the biofilm 24 h planktonic 24 h), which contained up-regulated DEGs 281, 382, 399, 271, 506 and down-regulated DEGs 250,356, 451, 328, 574, respectively. In **(A–E)**, the “triangles” represent the genes, the color “azure” is a significantly downregulated gene, the color “orange” is a significantly upregulated gene, and the color “gray” is a non-significant gene. **(F)** Venn diagram for overlaps of DEGs in **(A–E)**.

The molecular regulatory mechanisms of the differential genes are based on identifying the differential genes associated with specific phenotypes. There were 2624 (Figure 1A) and 2581 (Figure 1B) genes were detected in the Biofilm 5 h vs. Biofilm 10 h and biofilm 10 h vs. biofilm 24 h, respectively. There were 281 and 382 significantly upregulated genes and 250 and 356 significantly downregulated genes, respectively.

To identify the differential gene expression between the biofilm bacteria and planktonic bacteria at the same time, the differential gene analysis at 5 h (biofilm 5 h vs. planktonic 5 h), 10 h (biofilm 10 h vs. planktonic 10 h), and 24 h (biofilm 24 vs. 24 h) were analyzed at the same time for the differential gene analysis. The results showed indicated 2594 (Figure 1C), 2593 (Figure 1D), and 2592 (Figure 1E) genes expressing in the 5th h, 10th h, and 24th h, respectively, with significant upregulation of 399, 271, and 506 genes, and downregulation of 451, 328, and 574 genes, respectively.

The statistical analysis of the above five groups of DEGs (Figure 1F) revealed the simultaneous presence of 43 DEGs in the above five groups; 29 genes were common differential genes in the remaining four groups except for the biofilm 10 h vs. the planktonic 10 h group, with the *clfB* gene closely associated with the biofilm formation. The literature searches revealed 32 DEGs in the biofilm 5 h group, biofilm 10 h group, and biofilm 24 h group at the same time and were closely related to the biofilm formation with multiple genes related to the biofilm formation, such as *isdA, clfA, clfB, sdrE, atl,* and *isdD*. These common DEGs may play a significant role in forming biofilm serving as candidate genes for subsequent studies.

### Functional Annotation and Interaction Network Analysis of the Key Genes

**FIGURE 2:**
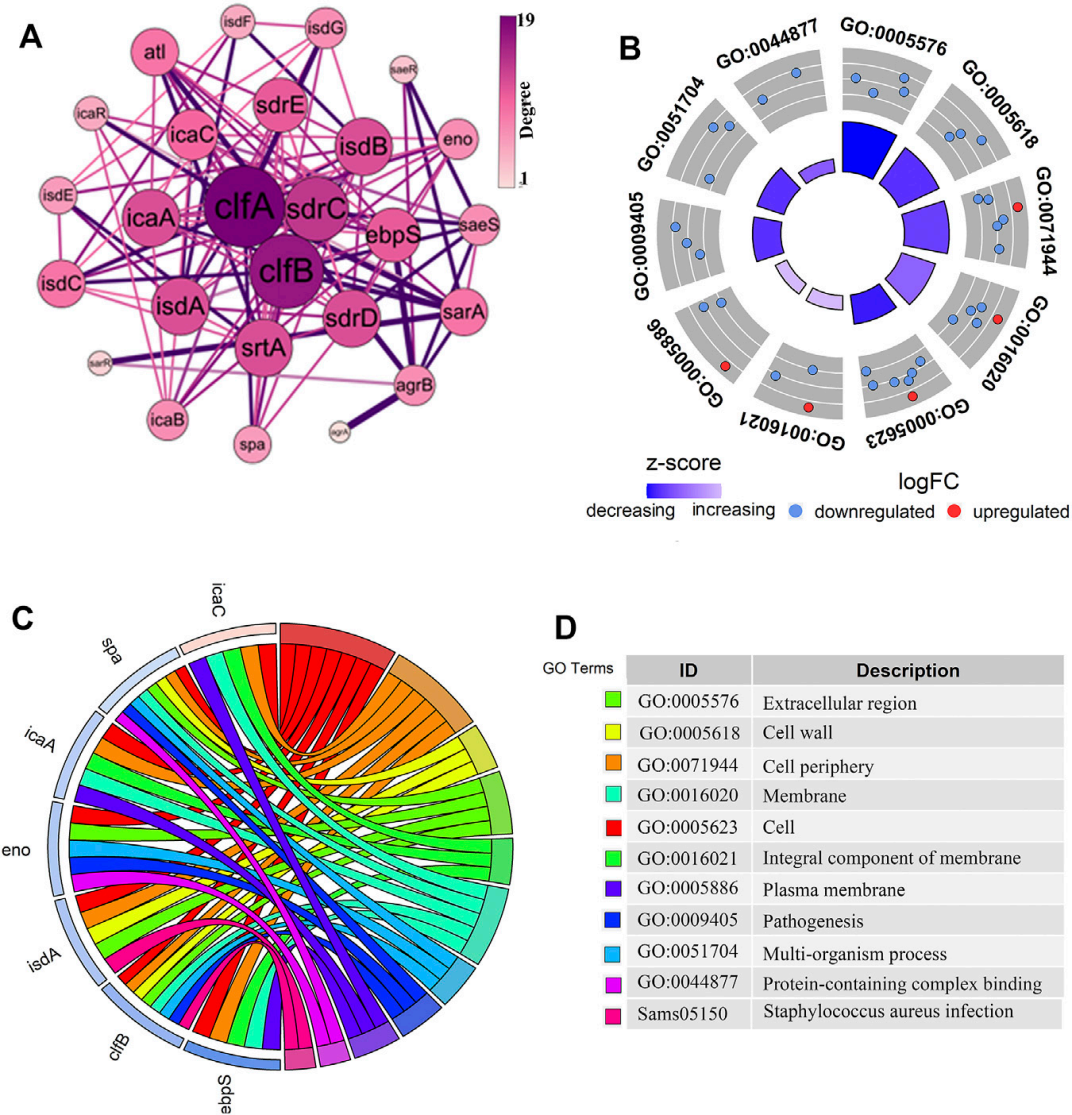
Analysis of the PPI network interactions and the functional enrichment of genes closely related to the biofilm formation. **(A)**. The PPI diagram of 32 genes related to biofilm formation, with the size and color intensity of the dotted circles representing the degree value of the genes. The larger circle with a darker color indicates a higher degree as well as a vital position in the network. **(B–D)**. The gene of interest enrichment analysis plots. The red and blue colors in **(B,C)** represented significantly upregulated and downregulated genes. The “z-score” represented the upregulated or downregulated status after centralization. The left color in **(D)** corresponds to the pathway enriched in **(C)**, and the proper annotation describes the terms in **(B,C)**.

We constructed a PPI network of differential genes (32 DEGs) associated with the biofilm formation was using the STRING website (Figure 2A), comprising 26 nodes representing the gene targets. The top ten genes were selected as the key candidate genes based on the ranking of the Degree values, arranged in descending order as *clfA, clfB, sdrC, isdA, isdB, sdrD, srtA, icaA, sdrE,* and *ebpS*. The gene function annotation helped understand the functions of these genes (Figures 2B–D). The GO results indicated these genes to be mainly enriched in the biological processes such as cell adhesion and pathogenicity. These genes were mainly localized in cellular fractions in the cell wall, extracellular region, cell periphery, septum, and intrinsic membrane proteins. The KEGG pathway enrichment analysis demonstrated these genes to be mainly involved in *S. aureus* infection. The functional annotation results revealed the role of these genes in biofilm formation through the relevant biological processes and signaling pathways and thus exerting the protection to *S. aureus* and the toxicity to the organism.

### Structural Characterization of the ClfB Protein

The differential analysis and enrichment analyses revealed a significant enrichment of the *clfB* gene in the pathogenesis and *S. aureus* infection pathways (Figure 2C). The network analysis showed that the *clfB* gene possesses an essential role in the PPI network (Figure 2A), and the expression (TPM) of *clfB* increased at 5, 10, and 24 h to 7.48, 8.51, and 10.16, respectively. The *clfB* may be a key gene for biofilm formation in *S. aureus*. Therefore, the *clfB* gene was selected as an essential candidate for this study.

**FIGURE 3:**
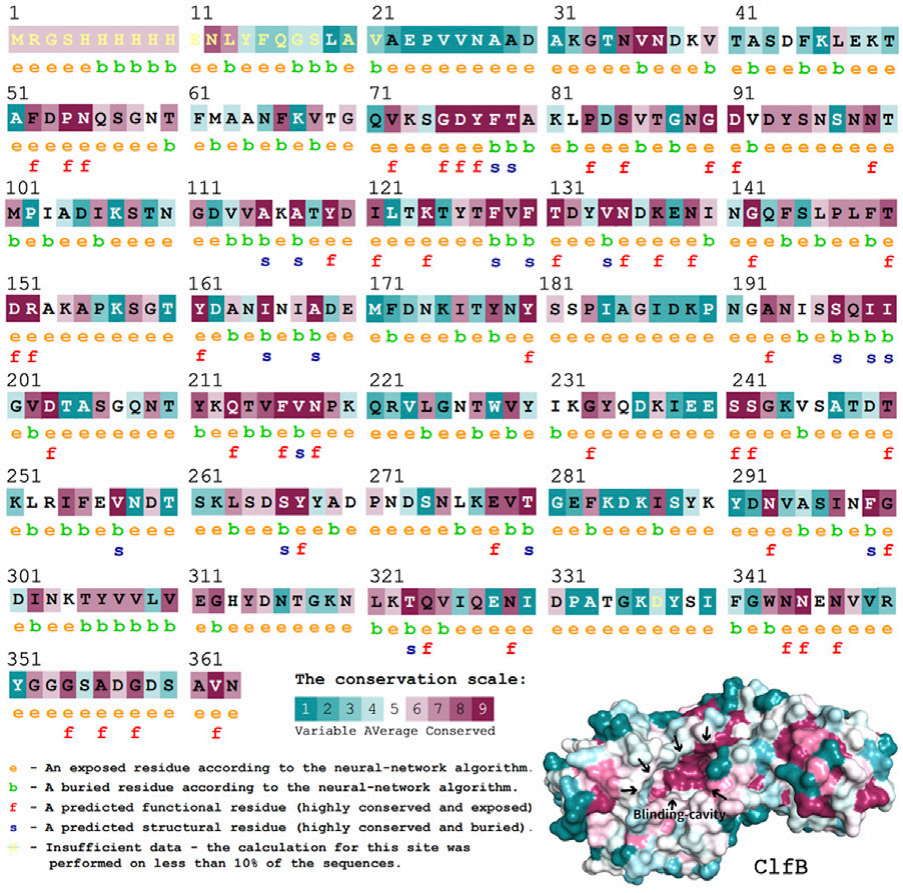
Structural prediction of ClfB protein, which contained the most secondary structures of *β*-folding (45.03%), followed by irregular coiling (36.65%), *β*-turning (11.49%), and *α*-helix (5.59%), and the binding cavity of ClfB comprised 20 amino acids, like L82, D84, D91, T100, D120, and G201.

It is fundamental to understand the secondary structures of the proteins translated to resolve their biological functions. As evident (Figure 3), the ClfB possesses the most secondary structures of *β*-folding (45.03%), followed by irregular coiling (36.65%), *β*-turning (11.49%), and *α*-helix (5.59%). The analysis of the convolutional neural networks showed 74.66% of amino acids to be located on the surface (exposed), and 25.34% of amino acids to be located inside (buried). In general, the conserved amino acids are located mainly on the surface of the protein structure, facilitating the protein’s biological function. On the other hand, the protein structure is stabilized by the amino acids located inside the protein. The ClfB proteins are highly conserved, exerting a solid and stable function, and the structure indicated that the binding cavity of ClfB comprises 20 amino acids, like L82, D84, D91, T100, D120, and G201. Among these 20 amino acids, six amino acid disabilities (D91, N99, Y119, I121, K124, and I200) are highly conserved. Meanwhile, the six amino acid residues (N89, D93, R152, I200, K212, E311) that contributed to the intermolecular forces are also highly conserved. The secondary and tertiary structures analysis showed that ClfB is highly conserved in the amino acid composition and is structurally stable, and these highly conserved amino acids form a binding cavity favorable for the other molecules to bind.

### Molecular Docking

**FIGURE 4:**
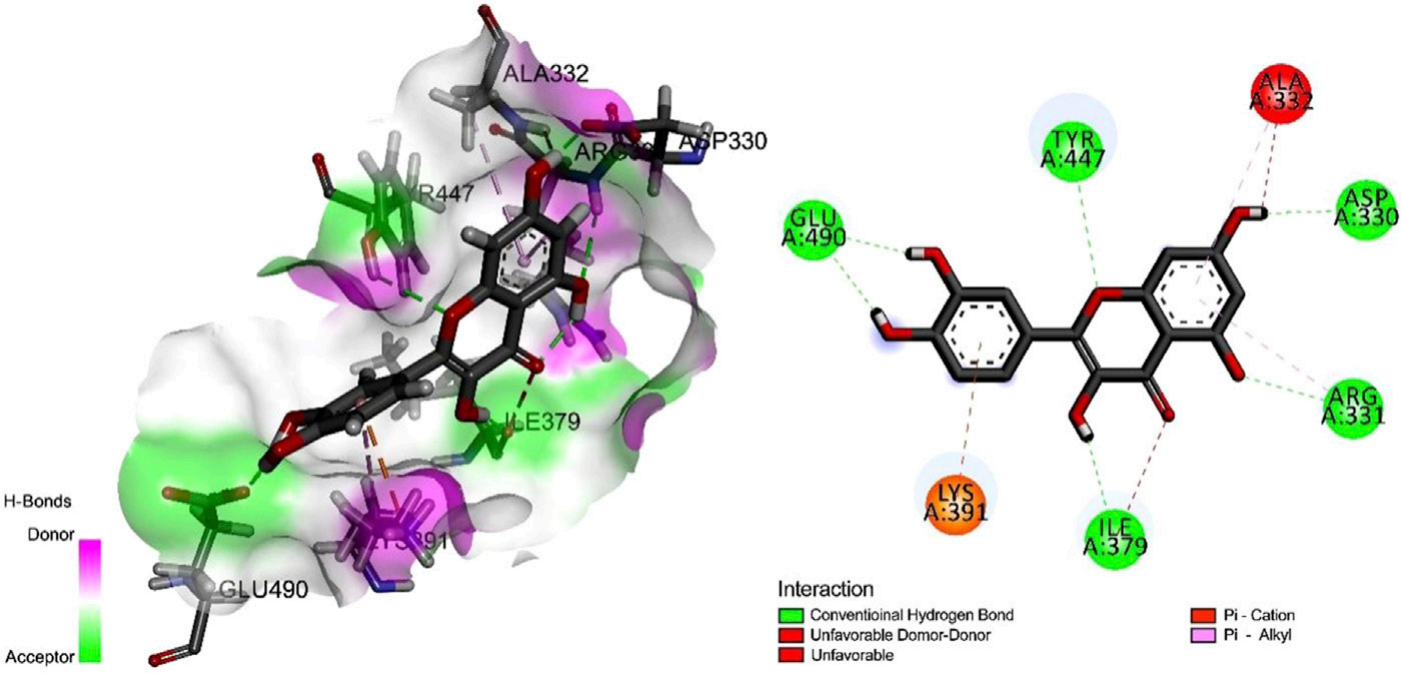
The molecular docking results of the ClfB protein and QEN, which showed that the QEN molecule form five H-bonds with the five amino acid residues (D330, R331, I379, Y447, and E490) of ClfB with a binding free energy of −7.7 Kcal/mol.

To determine the optimal docking site, the surface binding cavity of ClfB protein was predicted and analyzed based on the surface rolling ball model. The analysis revealed (Figure 4) multiple amino acid residues forming a deep cavity around (N268, G269, D272, D330, R331, A332, G380, K391, L488, E490), providing a binding site for QEN. The results of the docking studies showed that the QEN molecule form five H-bonds with the five amino acid residues (D330, R331, I379, Y447, and E490) of ClfB with a binding free energy of −7.7 Kcal/mol, suggesting that QEN could strongly bind and interact with ClfB proteins. The bound amino acids were distributed around the QEN molecule, enabling a more balanced force that stabilizes the QEN molecule in the binding cavity. The Pi-Alkyl intermolecular force compounded one benzene ring in the QEN with the amino acid residues R331 and A332. The docking experiments showed that the ClfB and QEN form a stable compound with more hydrogen bonds and lower binding energy. The Simulation trials showed that the stability of the ClfB binding pocket in the QEN-ClfB complex is primarily determined by residues D330, R331, I379, Y447, and E490.

### Molecular Dynamics Simulation

**FIGURE 5:**
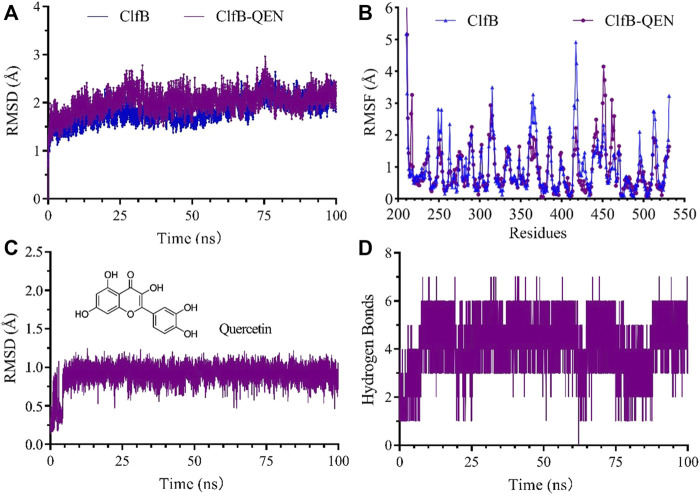
Results of the molecular dynamics simulation. **(A)** and **(B)** revealed that the ClfB-QEN compound system remained stable after convergence at 30 ns, and the compound's RMSD remained in the range of 1–3 Å with time and RMSF remained in the range of 0–2 Å among most amino acid residuals; **(C)** and **(D)** indicating that the high stability of the formed ClfB-QEN complex.

The higher the number of hydrogen bonds formed during the formation of the compound, the smoother is the degree of structural changes. Hence, we could suppose that the formed compound is stable. The analysis revealed that the ClfB-QEN compound system remained stable after convergence at 30 ns, and the compound’s root-mean-square deviation (RMSD) remained in the range of 1–3 Å with time (Figure 5A) and the root mean square fluctuation (RMSF) remained in the range of 0–2 Å among most amino acid residuals (Figure 5B). During the simulation (Figure 5D), the number of formed hydrogen bonds remained in the range of 3–6, with a maximum of seven hydrogen bonds, and only seven hydrogen bonds could be formed at 100 ns. This indicated that the high stability of the formed ClfB-QEN complex.

Figure 5C showed the dynamic analysis of QEN in the binding cavity where QEN does not induce a significant change in the binding cavity, indicating the docked conformation’s relative stability. To forecast the binding site of QEN to the ClfB protein, the contact rate analysis of the amino acids at the binding site was performed (Table 2). Combined with the molecular docking results, the residues N268, G269, D330, and I379 bound to QEN, were found to have a higher exposure frequency to the residues such as N234, D270, Y273, and F328, which are CK10 or Fg*α* interactions, and QEN binding would increase the volatility of the N234 affecting the binding of the ClfB protein to CK10 or Fg*α* to some extent.

**TABLE 2:** The exposure rate of the amino acid residue.

QEN and ClfB binding cavity site	CK10 and ClfB binding site	Fg*α* binding site with ClfB	Amino acid residue exposure rate
N268	Y273	Y273	0.76
I379	D270	D270	0.99
I379	N234	N234	0.954
G269	F328	F328	1.00
D330	N234	N234	0.884
D330	F328	F328	0.812
D330	D270	D270	0.972

### Effect of Quercetin on the Morphology of the *Staphylococcus aureus* Cells

**FIGURE 6:**
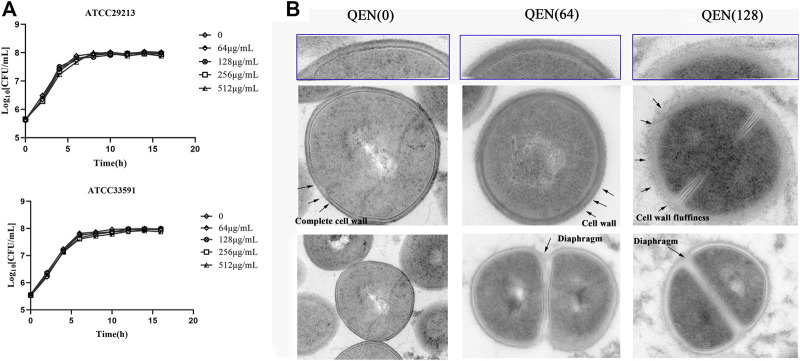
Effect of different concentrations of QEN on growth of the different bacterial strains *S. aureus*. **(A)** Effect of QEN on the growth of the two bacterial strains *S. aureus*, indicating that growth is not affected at the concentration used **(B)** Effect of QEN on the subcellular organelles of the strain WLD10, indicating that QEN counld trigger the sparseness of the *S. aureus* cell wall structure, but the division and proliferation.

To further investigate the effect of QEN on the growth ability of *S. aureus*, the growth curves of *S. aureus* were studied under the concentrations gradient of QEN (0 μg/ml, 64 μg/ml, 128 μg/ml, 256 μg/ml, 512 μg/ml). The results (Figure 6A) demonstrate that the MIC was higher than 512 μg/ml, and the growth trends of the bacteria strains in the two groups were almost similar to that of the control group. Thus, high concentrations of QEN were found only to delay the growth of planktonic bacteria but not inhibit the growth of planktonic bacteria. Secondly, the *MRSA-WLD10* cell wall structure changes were found by transmission electron microscopy, where the control group was found to have a uniform and neat bacterial morphology, with a round or oval shape and clear and neat cell wall boundary (Figure 6B). The addition of 64 μg/ml of QEN in the experimental group could make the cell wall edge of the WLD10 bacterial strain fluffy blurring the local outline. When the concentration reached 128 μg/ml, the cell wall persisted their thickness, the edges were roughened, and the outline was indistinguishable. However, the bacterial cells forming a standard diaphragm were evident in the field of view, indicating that QEN fails to inhibit bacterial diaphragm formation and normal bacterial replication. Even though the cell wall appeared abnormal, the cell division was not affected, which confirmed the results of the growth curve measurement. These results indicated the specific nature of the inhibitory effect of QEN on the *S. aureus* biofilm.

### The Quercetin Inhibition Assay on the Biofilm

**FIGURE 7:**
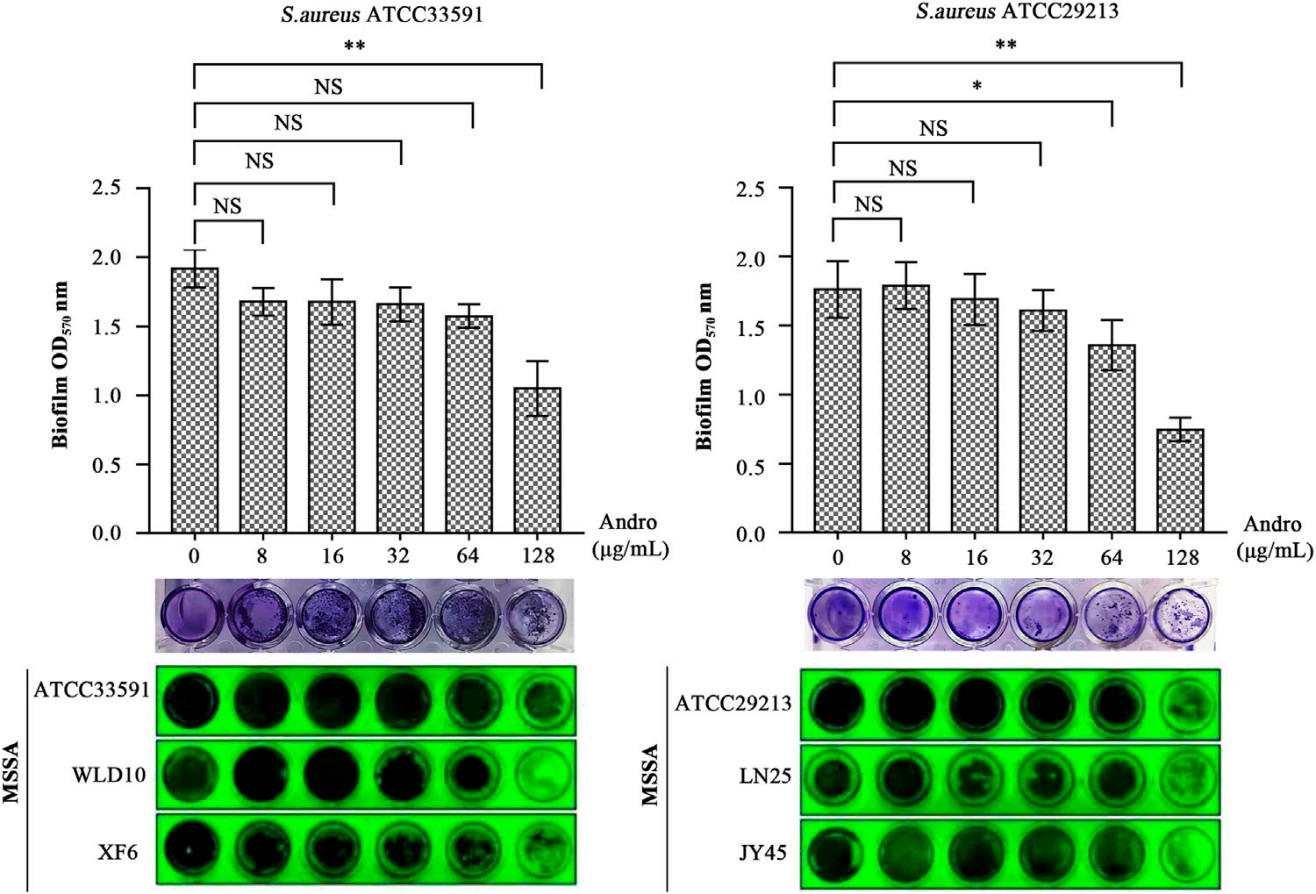
Inhibition of the *S. aureus* biofilm by QEN, indicating that QEN counld significantly inhibits biofilm formation when QEN concentration was 128 μg/ml.

As evident in Figure 7, QEN had a significant inhibitory effect on the formation of *S. aureus* biofilm compared to the control group (0 μg/ml). For bacterial strains ATCC33591, QEN at 128 μg/ml (MBIC) showed an extremely significant inhibitory effect on biofilm formation. For bacterial strains ATCC29213, the MBIC was 64 μg/ml. For both bacteria strains used, 128 μg/ml QEN had an extremely significant inhibitory effect on biofilm formation, more than 50% (*p* < 0.05). It is evident from Figure 7 that the inhibition of the *S. aureus* biofilm was more evident with the increase in the concentration of QEN. Therefore, QEN can be considered a potential anti-biofilm inhibitor.

## Discussion

The *S. aureus* biofilm formation is an essentially biological process for increasing *S. aureus* drug resistance and the virulence factors’ emission of the host. The biofilm establishment phase is an important period for producing the virulence factors (Costerton, 2012). *S. aureus* emits virulence factors into the host environment causing the relevant pathology. It also uses the biofilm to protect itself from phagocytosis by the drugs and host immune cells. QEN is a polyhydroxyflavonoid compound that has inhibitory effects on biofilm formation (Lee et al., 2013). Being widely present in plants, it can be a source of antibacterial compounds.

**FIGURE 8:**
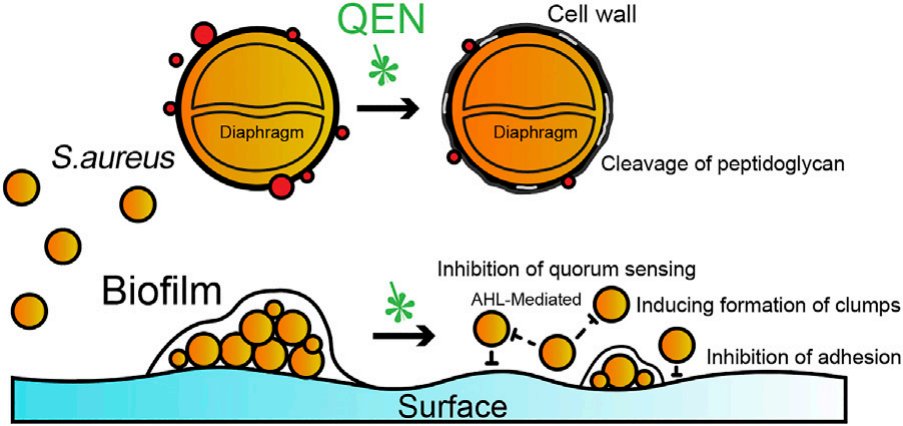
Potential schematic illustration of QEN inhibition on *S. aureus* and biofilms. The inhibition mechanism of QEN is bacterial colonization and adhesion by modulating quorum sensing.

QEN is reportedly capable of inhibiting the biofilms formation of many Gram-positive and negative bacteria. Available studies have shown that the primary effect of QEN on *S. aureus* is to inhibit bacterial colonization and adhesion by modulating quorum sensing, while having no direct killing effect on *S. aureus*. At MIC concentrations, QEN affected the morphology of the bacterial cell wall without affecting the normal growth of *S. aureus*, the process of which is summarised in Figure 8. In the two bacterial strains *S. aureus* studied (*MRSA* and *MSSA*, containing one standard and two isolates bacterial strain, respectively), QEN at 128 μg/ml could significantly inhibit biofilm formation. However, neither 64 μg/ml nor 128 μg/ml impaired the growth of *S. aureus*, which is consistent with the previous studies (Carrada and Sánchez, 2017; Wang et al., 2019). This differs from the results of Júnior et al., showing that 250–500 μg/ml QEN inhibited 50% of biofilm formation in the *MRSA* bacterial strains and is similar to the 50 μg/ml concentration of *MSSA ATCC 6538* (Lee et al., 2013; Júnior et al., 2018). To further determine whether QEN affects the growth of the *S. aureus*, the cell morphology of *S. aureus* was observed using transmission electron microscopy, and QEN was found to trigger the sparseness of the *S. aureus* cell wall structure, but the division and proliferation were not affected. QEN caused diverse degrees of roughness, dispersion and loosening of the *S. aureus* cell wall, indicating that QEN is able to affect cell wall turnover and metabolism; resulting in thickening of the aged peptidoglycan layer that is not removed in time. In addition, Tannic acid, a polyphenol similar to QEN, has shown an inhibitory effect on the biofilm (Lee et al., 2013), which is achieved by upregulating the protein levels of IsaA (immune-dominant staphylococcal antigen A), which divides the peptidoglycan layer of the bacterial cell wall. IsaA, a cleavage transglycosylase, has been proven to have the activity of cleaving peptidoglycan The cleavage process is similar to the cutting of the *β*-1,4 glycosidic bond (glycosidic bond) between N-acetylmuramic acid (MurNAc) and N-acetyl glucosamine (GlcNAc) by phage lytic enzymes. Neither QEN nor tannic acid inhibited bacterial growth during biofilm suppression, and the evident diaphragm was still formed normally. It is similar to previous studies that QEN does not impair the growth of *S. aureus* cells: when the QEN concentration was MIC (594 μg/ml), the membrane permeability was an imbalanced and intracellular protein and potassium ions were escaped (Perumal et al., 2017; Wang et al., 2019), and the membrane surface of *S. aureus* cells with extensive shrinkage and some degree of rupture (Feng et al., 2010). QEN was also found to reduce biofilm thickness and number of biofilm-encapsulated bacteria, increase biofilm permeability (Yan et al., 2018), which is similar to our observation that 128 mg/ml significantly reduced biofilm formation. These findings revealed that QEN could suppress biofilm formation without impairing the growth of *S. aureus*, indicating that treatment of biofilm infections with QEN would not exert selective pressure on *S. aureus* to induce drug resistance.

In the datasets we used, the expression of the *clfB* gene was found to differ significantly at 5, 10, and 24 h of biofilm formation, with a gradually increasing trend. The clfB protein is 150 kDa in length with an LPXTG sorting enzyme motif at the C-terminal end that covalently anchors the MSCRAMM (microbial surface component recognizing adhesive matrix molecule) to the cell wall peptidoglycan. The *clfB* gene expression has been found to increase during the growth of the biofilms (Resch et al., 2005), and clfB can mediate the biofilm formation (Abraham and Jefferson, 2012). Under the condition of Ca^2+^ ion exhaustion, when the *clfB* gene is knocked out, the biofilm growth can be significantly inhibited (Abraham and Jefferson, 2012). Meanwhile, ClfB can also bind to the fibrinogen activating it to form fibronectin and promoting agglutination, a process that plays an essential role in the second stage of biofilm formation. The regulator SpoVG is reportedly one of the widely regulated gene expression progenitors in *S. aureus*, which can directly bind to the *clfB* gene promoter region and positively regulate the expression of the *clfB* gene (Qing et al., 2018). The SpoVG gene expression in this study was 2.8-fold higher at 10 h (*p* < 0.05) and 1.8-fold higher at 24 h than at 10 h (*p* < 0.05). This also explains the continuous expression of the clfB gene with the growth cycle of biofilms from a regulatory perspective, which suggests an essential role for clfB in biofilm formation.

QEN could bind to the ClfB protein by forming five hydrogen bonds (D330, R331, I379, Y447, and E490) and six (N89, D93, R152, I200, K212, E311) to maintain the stability of the QEN-ClfB complex. To date, no studies have been reported on whether QEN could bind to ClfB proteins. Other studies have shown that QEN inhibits *S. aureus* infection by forming *π*-*π* bonds, van der Waals forces, and hydrogen bonds with residues Y187, L221, and H228 (K185) of coagulase (CoA), respectively (Wang et al., 2019; Gao et al., 2020); and by binding to the virulence factor alpha-toxin to reduce its virulence (Carrada and Sánchez, 2017). As there was no direct experimental evidence of QEN binding to ClfB protein in the present paper, we analyzed the exposure rate of the ClfB protein-binding cavity amino acid sites (N268, G269, D330, I379) to the active sites of CK10 and Fg*α* in the present paper. The exposure of ClfB to the active sites of CK10 or Fg*α* [N234, D270, Y273, and F328 (Walsh et al., 2004; Walsh et al., 2008)] was greater than 81% (except for N268, which was 76%) of the amino acid sites derived here. It was indicated that the ClfB protein binds to CK10, primarily contributing to the *S. aureus* colonization (Candi et al., 2005; Wertheim et al., 2008), and when the ClfB protein binds to Fg*α*, it promotes the transformation of fibrinogen to fibrin (Entenza et al., 2000) for aggregation. In addition to the evidence of higher exposure rates, a stable *π*-*π* bond may also be formed between Y447 and quercetin (Y187 forms a *π*-*π* bond with quercetin (Wang et al., 2019)). Therefore, the QEN-ClfB complex may develop a strong intermolecular force to stabilize this complex, and the formation of the QEN-ClfB complex may have changed the original molecular conformation (higher contact rate of QEN with the active sites of CK10 and Fg*α*), which in turns could regulates the infection of *S. aureus*.

Although the clustering factor ClfB was identified by the bioinformatics method, QEN was experimentally observed to inhibit the formation of the *S. aureus* biofilm. Also, QEN and ClfB were predicted to form a ClfB-QEN compound through the hydrogen or *π*-*π* bond, affecting the formation of the *S. aureus* biofilm by allosteric regulation. However, several studies are required to explore further that the QEN has a binding site with the ClfB proteins in *S. aureus*.

## Conclusion

The QEN-ClfB compound may bind by forming five hydrogen bonds and one *π*-*π* bond, leading to conformational changes in the adjacent active sites (binding sites of CK10 and Fg*α* to ClfB proteins), and thus influence bacterial colonization and adhesion to inhibit biofilm formation. We have speculated on the potential binding patterns of QEN and ClfB, but further experiments are required to validate their binding patterns, such as pullDown experiments, nuclear magnetic resonance, and crystal structure analysis. Overall, our results provide a new target for treating *S. aureus* infection—QEN may bind to ClfB protein to induce conformational changes in the adjacent active site, resulting in the inhibition of biofilm formation.

## Data Availability

Publicly available datasets were analyzed in this study. This data can be found here: GEO,GSE163153.

## Abbreviations

BP, biological process; CC, cellular component; CFU, colony forming units; DEGs, differentially expressed genes; FDR, false discovery rate; KEGG, Kyoto encyclopedia of genes and genomes; MF, molecular function; MHB medium, Mueller-Hinton broth; MIC, minimum inhibitory concentration; PDB, Protein data bank; PPI, the protein-protein interaction; QEN, quercetin; *S. aureus*, *Staphylococcus aureus*; RMSD, The Root Mean Square Deviation; RMSF, The Root Mean Square Fluctuation; TPM, Transcripts Per Kilobase of exon model per Million mapped reads.
